# The LINA Study: Higher Sensitivity of Infant Compared to Maternal Eosinophil/Basophil Progenitors to Indoor Chemical Exposures

**DOI:** 10.1155/2016/5293932

**Published:** 2016-05-26

**Authors:** Friederike Hörnig, Tibor Kohajda, Stefan Röder, Gunda Herberth, Martin von Bergen, Michael Borte, Ulrike Diez, Ulrike Rolle-Kampczyk, Jan-C. Simon, Judah A. Denburg, Irina Lehmann, Kristin M. Junge

**Affiliations:** ^1^Helmholtz Centre for Environmental Research (UFZ), Leipzig, Department of Environmental Immunology, Permoserstraße 15, 04318 Leipzig, Germany; ^2^Leipzig Research Center for Civilization Diseases (LIFE), University of Leipzig, Philipp-Rosenthal-Straße 27, 04103 Leipzig, Germany; ^3^Helmholtz Centre for Environmental Research (UFZ), Leipzig, Department of Metabolomics, Permoserstraße 15, 04318 Leipzig, Germany; ^4^Helmholtz Centre for Environmental Research (UFZ), Leipzig, Core Facility Studies, Permoserstraße 15, 04318 Leipzig, Germany; ^5^Helmholtz Centre for Environmental Research (UFZ), Leipzig, Department of Proteomics, Permoserstraße 15, 04318 Leipzig, Germany; ^6^Department of Biotechnology, Chemistry and Environmental Engineering, Aalborg University, Niels Jernes Vej 10, 9220 Aalborg East, Denmark; ^7^Children's Hospital, Municipal Hospital “St. Georg”, Academic Teaching Hospital of the University of Leipzig, Delitzscher Straße 141, 04129 Leipzig, Germany; ^8^University Hospital of Leipzig, Department of Dermatology, Venereology and Allergology, Philipp-Rosenthal-Straße 23, Haus 10, 04103 Leipzig, Germany; ^9^Department of Medicine, Division of Clinical Immunology and Allergy, McMaster University, 1200 Main Street West, Hamilton, ON, Canada L8N 3Z5

## Abstract

*Purpose*. Enhanced eosinophil/basophil (Eo/B) progenitor cell levels are known to be associated with allergic inflammation and atopy risk. The aim of the present study was to investigate the influence of different indoor exposures on the recruitment and differentiation of Eo/B progenitors in mother-child pairs.* Methods*. In 68 mother-child pairs of the LINA study peripheral blood mononuclear cells were used to assess Eo/B colony forming units (CFUs). Information about disease outcomes and indoor exposures was obtained from questionnaires. Indoor concentrations of volatile organic compounds (VOCs) were measured by passive sampling.* Results*. Infant's Eo/B CFUs were positively associated with exposure to tobacco smoke, disinfectants, or VOCs. In contrast, for maternal Eo/B CFUs, only a few associations were seen. Higher numbers of infant Eo/B CFUs were observed in children with wheezing symptoms within the second year of life.* Conclusions*. We demonstrate that infant's hematopoietic cells seem to respond with more sensitivity to environmental exposure compared to maternal cells. At least in infants, an activation of these hematopoietic cells by environmental exposure could contribute to an enhanced risk for the development of respiratory outcomes.

## 1. Introduction

Eosinophils and basophils are typical effectors of allergic inflammation [1]. In asthmatic patients, the numbers of eosinophils and basophils were found to be increased in tissue, blood, and bone marrow and also correlated with disease severity [1–3]. Additionally, several studies showed that eosinophil/basophil progenitor cells, which develop from bone marrow CD34^+^ cells under the influence of specific chemokines and cytokines [4], are increased in the peripheral blood of atopic subjects with asthma [5, 6], allergic rhinitis [6–8], nasal polyposis [8], or atopic skin manifestations [7] which was also reviewed in Denburg and Keith [9] and Gauvreau and Denburg [10]. In children, cord blood Eo/B progenitors differ in phenotype and function among infants at risk for atopy [11]. Cord blood progenitors were further seen to be predictive for frequency and characteristics of acute respiratory illness in infants during the first year of life [12]. Next to these quantitative relations to disease outcomes, Denburg et al. demonstrated that maternal diet during pregnancy can interfere with the number and function of progenitor cells and subsequently with disease outcomes such as atopic dermatitis and wheeze at one year of age [13]. In agreement, our own group contributed data showing that Eo/B progenitor cells of one-year-old children with cradle cap were increased in association with environmental pollutants [14]. So far, data showing a responsiveness of Eo/B progenitors to lifestyle and environmental factors are limited to the infant hematopoietic system. Within the LINA study (Lifestyle and Environmental Factors and Their Influence on Newborns Allergy Risk) we already demonstrated that cord blood but not maternal Th1/Th2 cytokine levels depends on chemical exposure during pregnancy [15]. This result may point to an increased vulnerability of infant compared to adult T cells to environmental exposure. The aim of the present study was to investigate whether allergy relevant eosinophil/basophil progenitor cells from infants also respond with more sensitivity to environmental pollutants compared to progenitor cells from their mothers living in the same environment.

## 2. Materials and Methods

### 2.1. Study Design

The mother-child study LINA was designed to investigate how environmental factors in the pre- and postnatal period influence the immune system and whether they are determinants of increased allergy risk later in children's life. For this study, 629 mother-child pairs (including 7 twins) were recruited between May 2006 and December 2008 in Leipzig, Germany. Mothers suffering from immune or infectious diseases during pregnancy were excluded from the study. Six hundred six mother-child pairs participated in the one-year and 546 pairs in the two-year follow-up (one scheduled visit/year around the child's birthday). Blood samples from 397 mothers and 340 children were obtained at children's age of two (mean age: 2 years and 26 days, min–max: 1 year and 343 days–2 years and 161 days) as part of the scheduled visit. Sufficient peripheral blood mononuclear cells (PBMCs) for methylcellulose cultures were available for a subset of 68 corresponding mother-child pairs (66 mothers, 68 children; 2 sets of twins). All relevant *N*-numbers are shown in Figure A.1 in Supplementary Data available online at http://dx.doi.org/10.1155/2016/5293932. Participation in the LINA study was voluntary and written informed consent was obtained from all participants. The study was approved by the Ethics Committee of the University of Leipzig (file ref. # 046-2006, 160-2008).

### 2.2. Questionnaire Data

Information concerning general aspects of life and environmental conditions during pregnancy was collected by detailed questionnaires during the 36th week of pregnancy. Further, information about respiratory outcomes of the child in the last 12 months as well as information about housing and environmental conditions (e.g., environmental tobacco smoke (ETS) exposure, and usage of cleaning agents) was obtained annually. All questionnaires were self-administered by the parents. For more detailed information, please see the methods section of Supplementary Data.

### 2.3. Preparation of Peripheral Blood Samples

PBMCs were isolated within six hours after collection of about 3 mL (child) to 5 mL (mother) fresh heparinised peripheral blood via Ficoll Paque density centrifugation. PBMCs were frozen in 1 mL aliquots of 90% fetal bovine serum and 10% dimethylsulfoxide with 10–30 × 10^6^ cells. The cell thawing protocol and isolation of nonadherent mononuclear cells (NAMNCs) were performed according to Reece et al. [16]. Viability of PBMCs after thawing and NAMNCs after 2 h incubation averaged 93.4%.

### 2.4. Methylcellulose Cultures

In the present paper a well-established and prevalidated method of functional methylcellulose assay was used to assess Eo/B CFUs [12, 14, 16]. To assess eosinophil/basophil progenitor cell differentiation by colony formation 5 × 10^5^ NAMNCs were incubated in duplicate in the presence of recombinant human cytokine IL-3 (1 ng/mL), IL-5 (1 ng/mL), or GM-CSF (10 ng/mL) (R&D Systems Europe Ltd., Abingdon, Oxon, UK) over 14 days. Eosinophil/basophil colony forming units (CFUs) were detected and enumerated by their characteristic morphology using a light inverted microscope. Details were described earlier [12, 14, 16]. The investigator, who performed the progenitor assays and analyses, was blinded to the identity of subjects and other data collected. Due to insufficient cell numbers after thawing, methylcellulose assays could not be performed for all three cytokines in certain samples. Case numbers for IL-3-, IL-5-, and GM-CSF-stimulated cultures resulted in a total of 67, 66, and 56 analysable samples for the children and in 63, 36, and 11 samples for the mothers, respectively.

### 2.5. Measurement of VOC and Cotinine Concentrations

To measure the individual exposure to volatile organic compounds (VOCs) in the homes, passive samplers (3M monitors, type OVM 3500; 3M GmbH, Neuss, Germany) were placed in the child's bedroom (or alternatively in the room where the child preferentially spent most of their time) around the first birthday of the child. Concentrations of VOCs were analysed as described earlier [17] and adjusted for seasonal variations as described in Schlink et al. [18]. For analyses of cotinine see the methods section of the Supplementary Data.

### 2.6. Statistical Analyses

Statistical tests were performed using STATISTICA for Windows Version 10.0 (StatSoft Inc. Europe, Hamburg, Germany). The chi squared test was performed to compare parameters of the analysed subcohort with the remaining LINA cohort (*N*: 546 − 68 = 478).

Analyses related to Eo/B CFUs are calculated within the described subcohort (*N* = 68 mother-child pairs, including two sets of twins). Calculations for general associations independent of Eo/B CFU analyses (e.g., ETS exposure versus VOC concentrations) were performed for the entire LINA cohort (*N* = 546). When statistical analyses required a division into subgroups (e.g., exposure to ETS: yes versus no) data were not presented for *N*-numbers <5.

It was tested whether the available sample size for Eo/B analyses has the power to detect expected effect of indoor pollutants (in particular VOCs). With a type one error rate (alpha) of 0.05, a power goal of 0.9, and an expected population correlation of 0.460 (according to our earlier paper [14] the Eo/B CFUs and the total sum of all VOCs correlated with *R* = 0.460), the required sample size for such analyses was 45.

Since the majority of parameters were not normally distributed, analyses were performed using nonparametric tests in general. To address the relationship between the numbers of Eo/B CFUs and atopic outcomes or exposure to indoor ETS or disinfectants, medians were compared using the Mann-Whitney *U* test. To verify these results, multiple logistic regression models were used to determine adjusted odds ratios (OR) with a 95% confidence interval. The following confounders were used: month of birth (for seasonal changes), gender of the child (for potential differences between boys and girls), parental school education (as a marker for the social status), and family history of atopy (to consider the individual genetic background), as well as exposure to indoor ETS during pregnancy, maternal cotinine level at the child's first birthday, the sum of all measured VOCs at the child's first birthday (both cotinine and VOCs as a marker for ETS exposure in the second year of life), dampness in the dwelling during second year of life (as a marker for early airway allergen exposure), and children with infections of the airways in the second year of life (to consider a potential nonallergic origin of wheezing). Spearman's rank correlation test was applied to analyse the association between maternal and infant number of Eo/B CFUs and between the number of smoked cigarettes and Eo/B CFUs as well as between VOC concentrations and the number of Eo/B CFUs. All *p* values <0.05 were considered to be significant. Adjustments due to multiple testing were not performed since our analyses were based on an* a priori* hypothesis [19].

## 3. Results

### 3.1. Characteristics of the Study Population

Characteristics of the study population are shown in Table 1(A). There were no differences in the distribution of considered parameters in the analysed subcohort (*N* = 68) compared with the remaining LINA cohort (*N* = 478).

**Figure 1:**
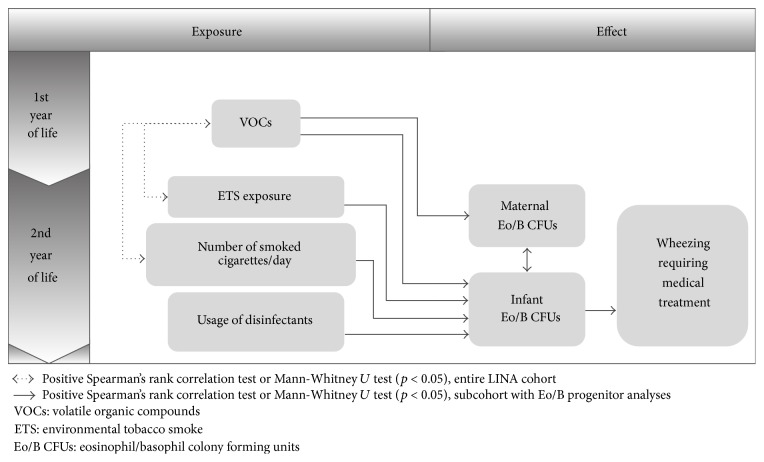
Associations among indoor chemical exposures, Eo/B CFUs of mother-child pairs, and clinical outcomes of 2-year-old children.

In general, a positive correlation was found between corresponding maternal and infant IL-3- (*p* < 0.001, *R* = 0.447, *N* = 65) as well as GM-CSF- (*p* = 0.028, *R* = 0.686, *N* = 10) stimulated Eo/B CFUs (Table 2). All shown significant associations between Eo/B CFUs, environmental exposures, and clinical outcomes are summarized in Figure 1.

### 3.2. Indoor Chemical Exposures: Disinfectants, ETS, and VOCs

During the second year of life 61.5% of the families in the analysed subcohort declared their usage of disinfectants in the household as “once a week or more,” “once a month or more,” or “occasionally,” with no differences when compared to the remaining LINA cohort (*p* = 0.608, Table 1(B)).

Within the analysed subcohort, 10.4% of the participants were exposed to indoor ETS “(almost) daily,” “once a week or more,” or “occasionally,” with no differences when compared to the remaining LINA cohort (*p* = 0.222, Table 1(B)). ETS exposed mothers had higher urine cotinine levels (median: 42.15 *μ*g/g, IQR: 2.02–308.30) compared to mothers without ETS exposure (median: 1.22 *μ*g/g, IQR: 0.43–3.92; *p* < 0.001). Further, a positive correlation between maternal urine cotinine levels and the number of smoked cigarettes per day in the dwellings was observed (*p* < 0.001, *R* = 0.171). Smoking at home was related to enhanced indoor concentrations of the aromatic VOCs benzene (*p* = 0.002) and m + p-xylene (*p* = 0.048). In addition the number of smoked cigarettes per day was correlated with indoor benzene concentrations (*p* = 0.008, *R* = 0.117).

### 3.3. Association between Eo/B CFUs and Indoor Chemical Exposure

Two-year-old children exposed to ETS at home had significant higher numbers of IL-3- (*p* = 0.010) and GM-CSF-stimulated Eo/B CFUs (*p* = 0.014, Table 3 and Supplementary Table A.1) compared to children without ETS exposure. Coincident with these findings, children's IL-3- and GM-CSF-stimulated Eo/B CFUs correlated positively with the number of smoked cigarettes per day (Table 4). In contrast, maternal IL-3-stimulated Eo/B CFUs showed no association either with ETS exposure at home or with the number of smoked cigarettes per day.

With respect to VOCs, positive correlations were found between children's IL-3-, IL-5-, or GM-CSF-stimulated Eo/B CFUs and the sum of all measured VOCs as well as for the single smoking related VOCs benzene, m + p-xylene, and o-xylene (*p* < 0.05, Table 5). In mothers, only one correlation was seen for IL-3-stimulated Eo/B CFUs and benzene.

The usage of disinfectants was found to be associated with increased numbers of GM-CSF-stimulated Eo/B CFUs among infants (*p* = 0.031, Table 3), while maternal Eo/B CFUs did not vary significantly.

### 3.4. Respiratory Outcomes

Within the analysed subcohort, 22.7% of the children were positive for wheezing ever, 19.0% for recurrent wheezing, and 10.3% for wheezing requiring medical treatment during the second year of life. Furthermore a physician-diagnosed bronchitis was seen in 28.6% of the children and obstructive bronchitis in 9.4%. There were no differences in the distribution of considered respiratory outcomes in the analysed subcohort (*N* = 68) compared to the remaining cohort (*N* = 478, *p* > 0.05, Table 1(C)).

### 3.5. Association between Infant Eo/B CFUs and Respiratory Outcomes during the Second Year of Life

**Figure 2:**
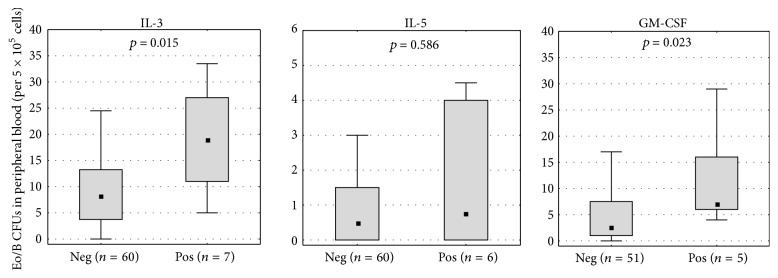
IL-3-, IL-5-, or GM-CSF-stimulated Eo/B CFUs in peripheral blood of two-year-old children with (pos) or without (neg) wheezing requiring medical treatment during the second year of life. Data are shown as box plots with median and 25th to 75th percentile. *p* values < 0.05 are considered to be significant (Mann-Whitney *U* test).

Children who suffered from wheezing requiring medical treatment during the second year of life had significantly more IL-3- (*p* = 0.015) and GM-CSF-stimulated Eo/B CFUs (*p* = 0.023, Figure 2) at the age of two. The association between IL-3-stimulated Eo/B CFUs and wheezing with medical treatment remains stable after adjustment for possible confounding factors (month of birth, gender of the child, parental school education, family history of atopy, and exposure to indoor ETS during pregnancy as well as maternal cotinine level, the sum of all measured VOCs, dampness, and infections of the airways in the second year of life). We considered airway infections as an additional factor since wheezing episodes can also occur together with airway (and in particular virus) infection. No significant association was found between Eo/B CFUs and the occurrence of wheezing symptoms ever (IL-3: *p* = 0.926; IL-5: *p* = 0.379; GM-CSF: *p* = 0.943), recurrent wheezing (IL-3: *p* = 0.574; IL-5: *p* = 0.415; GM-CSF: *p* = 0.909), bronchitis (IL-3: *p* = 0.281; IL-5: *p* = 0.067; GM-CSF: *p* = 0.095), or obstructive bronchitis (IL-3: *p* = 0.308; IL-5: *p* = 0.495; GM-CSF: *p* = 0.663).

## 4. Discussion

Within earlier studies, we showed that there are increases in blood eosinophil/basophil progenitor cells in one-year-old children in association with exposure to environmental chemicals [14]. In the current work we wanted to clarify whether this progenitor cell responsiveness is specific to the infant hematopoietic system or can also be seen in adults. Therefore, we analysed mother-child pairs living under the same environmental conditions for their differentiation of Eo/B progenitor cells.

To our knowledge absolute Eo/B colony numbers of mothers and their infants have not been compared before. It is well known that numbers of progenitor cells, except in bone marrow, are highest in cord blood and decrease in peripheral blood later in life [20]. Our data suggest that the absolute number of eosinophil/basophil progenitor cells in peripheral blood of two-year-old children is already comparable to adult levels, either when compared with their own mothers or with peripheral blood samples from former studies [21].

According to our hypothesis we could demonstrate with the present data that infant's progenitor cells seem to respond with more sensitivity to environmental pollutants (ETS, VOCs, and disinfectants) compared to maternal progenitor cells. This is in agreement with results shown earlier within the LINA study: VOCs emitted due to renovation activities were observed to influence the child's but not the mother's immune response. In cord blood but not in peripheral blood of the mothers increased IL-4 and IL-5 serum levels [15] were seen in relation to chemical exposure due to renovation activities during pregnancy. The current study provides further evidence that under similar exposure scenarios the infant's immune system is more susceptible to the influence of environmental exposure compared to the maternal immune system. We hypothesize that this increased sensitivity goes back to the still not fully mature infant's immune system. Compensation mechanisms which might lower/negate the adverse effects of environmental exposure in adults might not yet be fully developed in young children.

In addition, our data support results showing that enhanced numbers of Eo/B progenitor cells as a consequence of environmental pollutants may increase the risk for wheeze and skin manifestations in early infancy [13, 14]. However, these earlier studies based their findings of lifestyle- or environment-dependent differentiation of Eo/B progenitors on a selected high-risk study population [13, 14]. In the present paper we provide strong evidence that the impact of environmental pollutants on stem cell differentiation is also seen in the general population.

The fact that exposure to tobacco smoke/VOCs seems to influence the development of respiratory diseases has been shown before. There are several epidemiological studies demonstrating that exposure to prenatal smoke or passive tobacco smoke early in life is associated with an increased risk of wheezing in early infancy [22–26]. For example, passive household smoke exposure enhanced the risk for wheeze in children ≤2 years of age (OR = 1.35) [22]. Similarly, Pattenden et al. [25] demonstrated that smoking exposure within the first 2 years of life was associated with increased risk for wheeze (OR: 1.17).

There is furthermore evidence that exposure to tobacco smoke/VOCs provokes an immunological imbalance. Newborn children from smoking mothers were reported to have fewer cord blood regulatory T cell numbers [27] and an enhanced susceptibility to microbial infections through alterations of toll-like receptor- (TLR-) signalling [28] as well as a weak Th1 stimulation capacity [29]. As demonstrated in an earlier study by our group, exposure to VOCs may also direct the child's immune system towards a Th2 phenotype [17, 30]. A Th2 milieu caused by external stimuli has been shown to induce trafficking of IL-5-producing Th2 lymphocytes to the bone marrow where they promote eosinophilopoiesis through IL-5R signalling [11, 31]. In addition, the Th2 cytokines like IL-4 and IL-13 were described to regulate the transmigration of eosinophils from bone marrow to the tissues [32]. In children with skin manifestations a correlation between IL-4 blood levels and stimulated Eo/B progenitor cells was reported [14]. Thus, considering the fact that environmental pollutants such as tobacco smoke or VOCs induce a Th2 response in the child, we hypothesize that this may favour Eo/B progenitor cell differentiation, which could in turn contribute to an enhanced development of respiratory outcomes.

Finally, the present data demonstrate that Eo/B progenitors of two-year-old children which correlated positively with wheezing in early childhood were progenitors stimulated by IL-3 and GM-CSF: cytokines which are known to influence early eosinophil/basophil lineage differentiation [12]. Fernandes et al. [12] also showed that IL-3- and GM-CSF-stimulated Eo/B CFUs in cord blood are predictive for acute respiratory illnesses with fever or wheeze in the first year of life. This group and others discussed the hypothesis that immature progenitors are key determinants of atopic risk [10, 12]. Our data confirm this hypothesis by showing a correlation between severe wheeze (requiring medical treatment) and early-stage Eo/B CFUs in two-year-old infants. Furthermore, within the LINA study, we have also found a positive association between Eo/B progenitor cells in cord blood and respiratory outcomes during the first two years [33]. We could not include data on asthma in our paper, since asthma prevalence at this age is comparably low. However, wheezing may favour asthma development later in life: it was shown, for example, that wheeze present in high-risk infants may be transient or remain persistent through childhood. It was also suggested that 15% of infants who wheeze progress to chronic asthma, mostly those associated with family history of atopy [34].

The strength of our study derives from the fact that the analyses of Eo/B progenitor cells were performed in mother-child pairs which are well characterised regarding their immune parameters, atopic outcomes, and indoor air exposure to environmental chemicals. For example, individual ETS exposure was assessed not only by questionnaire data, which are always dependent on honesty and compliance of the participants [35], but in addition by analysis of maternal urine cotinine levels and moreover the measurement of VOC concentrations in the homes. All of these parameters highly support each other and represent an objective ETS exposure scenario, which shows a consistent positive association with infant Eo/B CFUs. By coupling progenitor cell measurements with environmental exposures and disease outcomes we were able to address the question of possible mechanisms responsible for the environmentally triggered increase in allergic outcomes.

A weakness of the LINA study in general is the potential bias by high rates of participating atopic parents (about 65%). We addressed this point by including family history of atopy as a confounding variable in the regression models. The high prevalence of atopic parents (who are already aware of their atopic disease and probably avoid potential hazards more than others) might also be one reason for the quite low prevalence of children exposed to ETS during the second year of life (10% versus 18.7% shown earlier for Germany [25]). One other limitation of the LINA study is that measurements of VOC and cotinine concentrations are only available at the child's first birthday due to missing home visits in the second year of life. However, we could demonstrate an almost consistent smoking behaviour of the study participants within the first and second year of life (85.7%). Therefore, we assume that VOC and cotinine levels measured at the child's first birthday also represent the child's exposure around the second birthday. This was confirmed by significant associations between ETS exposure within the second year of life and VOC or cotinine concentrations measured at the child's first birthday. Another limitation of the study is the restricted number of cases with stem cell analyses due to the very high experimental effort resulting in low numbers of children in certain outcomes. However, to our knowledge, the number of Eo/B progenitor cell analyses included in the present paper (in total almost 140) is higher than in any other earlier published study focused on health effects in relation to Eo/B progenitor cell function. Also some conditions of stimulated Eo/B progenitor cells (especially maternal IL-5 and GM-CSF CFU) resulted in small numbers of cases due to low number of available PBMCs. This might reduce the strengths of the reported results. Therefore, the presented results have to be interpreted with caution and need further validation.

## 5. Conclusions

In the present study we could confirm our earlier published data [14] showing that infant Eo/B progenitor cell differentiation is associated with indoor chemical exposure. Therefore, at least in infants, an increase of these hematopoietic cells by environmental exposure could contribute to an enhanced risk of the development of respiratory outcomes. The association of indoor chemical exposure and the differentiation of Eo/B progenitors appears to be mainly restricted to the infant's hematopoietic system. This is consistent with earlier results from the LINA study showing that cord blood but not maternal Th1/Th2 cytokine levels depends on chemical exposure during pregnancy [15]. Taken together, we can state that children's immune and hematopoietic cells seem to be more sensitive to environmental exposure compared to maternal cells. These results further support the hypothesis of a highly vulnerable and exposure sensitive time window in the perinatal period with consequences for children's disease risk. Protection against harmful environmental exposure and lifestyle conditions is therefore of much higher relevance for young children compared to adults. However, data needs to be confirmed in a larger cohort to verify the results based on the present small sample size.

## Supplementary Material

Supplementary data provide detailed information about the data collection by questionnaires according to respiratory outcomes as well as lifestyle and environmental conditions. Further, the measurement of urinary cotinine concentration is described. Table A.1 provides relevant case numbers for analyses of indoor chemical exposures and Eo/B CFUs of mothers and infants. Figure A.1 provides an overview about resulting N-numbers for Eo/B CFU analyses within the LINA study.

## Figures and Tables

**Table 1:** Characteristics of the analysed subcohort and the remaining LINA cohort. (A) General characteristics assessed during the 36th week of pregnancy. (B) Indoor chemical exposures during the second year of life. (C) Infant's respiratory outcomes during the second year of life.

Parameters	Analysed subcohort *n* (%), *N* = 68^*∗*^	Remaining cohort *n* (%), *N* = 478^†^	*p* value^‡^
(A) General characteristics			
** **Month of birth			
** **Nov–Feb	16 (23.5)	140 (29.3)	0.502
** **Mar-Apr, Sept-Oct	28 (41.2)	162 (33.9)	
** **May–Aug	24 (35.3)	176 (36.8)	
** **Gender of the child			
** **Female	29 (42.6)	235 (49.2)	0.349
** **Male	39 (57.4)	243 (50.8)	
** **Parental education^§^			
** **Low	0 (0)	6 (1.3)	0.294^*∗∗*^
** **Intermediate	19 (27.9)	101 (21.1)	
** **High	49 (72.1)	371 (77.6)	
** **Family history of atopy^‖^			
** **Double positive	18 (26.5)	80 (16.7)	0.167
** **Single positive	26 (38.2)	234 (49.0)	
** **Negative	24 (35.3)	164 (34.3)	
** **Exposure to ETS in dwelling during pregnancy^¶^			
** **Yes	11 (16.2)	59 (12.3)	0.430
** **No	57 (83.8)	419 (87.7)	
Dampness in dwelling during the second year of life			
** **Yes	6 (9.2)	46 (10.9)	0.700
** **No	59 (90.2)	377 (89.1)	

(B) Indoor chemical exposure during the second year of life			
** **Exposure to ETS in dwelling^¶^			
** **Yes	7 (10.4)	27 (5.7)	0.222
** **No	60 (89.6)	445 (94.3)	
** **Number of smoked cigarettes/day in dwelling			
** **≥15	3 (4.5)	7 (1.5)	0.173^*∗∗*^
** **1–14	4 (6.0)	10 (2.2)	
** **0	60 (89.5)	447 (96.3)	
Usage of disinfectants			
** **Yes	40 (61.5)	294 (65.0)	0.608
** **No	25 (38.5)	158 (35.0)	

(C) Respiratory outcomes during second year of life			
** ** Wheezing ever			
** **Positive	15 (22.7)	101 (21.8)	0.878
** **Negative	51 (77.3)	362 (78.2)	
** **Recurrent wheezing			
** **Positive	12 (19.0)	54 (12.5)	0.207
** **Negative	51 (81.0)	377 (87.5)	
** **Wheezing requiring medical treatment			
** **Positive	7 (10.3)	26 (5.7)	0.231
** **Negative	61 (89.7)	432 (94.3)	
** **Bronchitis			
** **Positive	18 (28.6)	125 (27.7)	0.887
** **Negative	45 (71.4)	326 (72.3)	
** **Obstructive bronchitis			
** **Positive	6 (9.4)	42 (9.6)	0.962
** **Negative	58 (90.6)	396 (90.4)	

^*∗*^ *N* may be different from 68 due to missing data.

^†^ *N* may be different from 478 due to missing data.

^‡^Calculated using the chi squared test for cross relationship.

^§^Low = 9 yrs of schooling or less “Hauptschulabschluss”; intermediate = 10 yrs of schooling “Mittlere Reife”; high = 12 yrs of schooling or more “(Fach-)hochschulreife.”

^‖^Family history of atopy is defined as occurrence of asthma, atopic dermatitis, hay fever, or food allergy.

^¶^Yes = (almost) daily, once a week or more, or occasionally; no = never.

^*∗∗*^Calculated although *n* numbers are <5 in some subgroups.

**Table 2:** Numbers of maternal and infant IL-3-, IL-5-, or GM-CSF-stimulated eosinophil/basophil (Eo/B) colony forming units (CFUs), presented as median and interquartile range (IQR). Correlation between maternal and infant Eo/B CFUs was calculated using Spearman's rank correlation test; *p* values < 0.05 are considered to be significant and printed in bold.

Eo/B CFUs	Mother	Child	*p*	*R*
	Median (IQR)			
IL-3	8.5 (3.5–17.5)	9.0 (4.0–15.0)	**<0.001**	0.447
IL-5	0.5 (0.3–1.5)	0.5 (0–1.5)	0.565	0.101
GM-CSF	5.5 (1.0–7.0)	2.5 (1.0–8.3)	**0.028**	0.686

**Table 3:** Association between indoor chemical exposures and the number of IL-3-, IL-5-, or GM-CSF-stimulated eosinophil/basophil (Eo/B) colony forming units (CFUs) of two-year-old children and their mothers. Data are shown as median and interquartile range (IQR); analyses were performed using Mann-Whitney *U* test; *p* values < 0.05 are considered to be significant and printed in bold.

Indoor exposures	Exposure to ETS in dwelling			Usage of disinfectants in the household		
	Yes	No	*p*	Yes	No	*p*
Eo/B CFUs	Median (IQR)			Median (IQR)		
Mother						
IL-3	18.8 (5.0–55.5)	8.5 (3.8–17.0)	0.116	10.5 (4.0–16.5)	7.8 (4.0–17.5)	0.790
IL-5			*∗*	0.5 (0.3–1.3)	1.0 (0.5–1.5)	0.597
GM-CSF			*∗*	7.0 (5.5–7.0)	4.8 (1.0–5.5)	0.312
Child						
IL-3	23.0 (9.5–32.0)	8.0 (3.5–14.0)	**0.010**	9.8 (4.8–19.8)	6.0 (3.0–12.5)	0.067
IL-5	1.0 (0.5–4.5)	0.5 (0–1.5)	0.100	0.5 (0–1.8)	0.5 (0–1.5)	0.930
GM-CSF	12.0 (3.0–16.0)	2.5 (1.0–6.5)	**0.014**	3.8 (2.0–12.3)	1.8 (1.0–5.5)	**0.031**

*∗* Reduced number of cases; see Table A.1 of the Supplementary Data.

ETS: environmental tobacco smoke.

**Table 4:** Correlation of indoor smoked cigarettes per day and IL-3-, IL-5-, or GM-CSF-stimulated eosinophil/basophil (Eo/B) colony forming units (CFUs) in peripheral blood of two-year-old children and their mothers. Data are shown as Spearman's rank correlations; *p* values < 0.05 are considered to be significant and printed in bold.

	Eo/B CFUs mother						Eo/B CFUs child					
	IL-3		IL-5		GM-CSF		IL-3		IL-5		GM-CSF	
	*p*	*R*	*p*	*R*	*p*	*R*	*p*	*R*	*p*	*R*	*p*	*R*
Number of smoked cigarettes/day in dwelling	0.131	0.194	*∗*	*∗*	*∗*	*∗*	**0.007**	**0.330**	0.096	0.208	**0.011**	**0.339**

*∗* Reduced number of cases; see Table A.1 of the Supplementary Data.

**Table 5:** Correlation of indoor volatile organic compound (VOC) concentrations and IL-3-, IL-5-, or GM-CSF-stimulated eosinophil/basophil (Eo/B) colony forming units (CFUs) in peripheral blood of two-year-old children and their mothers. Shown are aromatic VOCs as well as the sum of all analysed VOCs. Data are presented as Spearman's rank correlations; *p* values < 0.05 are considered to be significant and printed in bold.

VOCs	Eo/B CFUs mother						Eo/B CFUs child					
	IL-3		IL-5		GM-CSF		IL-3		IL-5		GM-CSF	
	*p*	*R*	*p*	*R*	*p*	*R*	*p*	*R*	*p*	*R*	*p*	*R*
Benzene	**0.029**	**0.275**	0.076	0.299	0.544	0.206	0.101	0.202	**0.036**	**0.258**	**0.036**	**0.281**
Toluene	0.899	0.016	0.834	0.036	0.779	0.096	0.750	0.040	0.314	0.126	0.433	0.107
m + p-Xylene	0.872	0.021	0.957	0.009	0.447	0.256	0.418	0.101	**0.006**	**0.332**	**0.017**	**0.318**
o-Xylene	0.483	0.090	0.882	−0.026	0.472	0.243	0.174	0.168	**0.005**	**0.345**	**0.002**	**0.407**
Styrene	0.777	0.037	0.282	−0.184	0.728	0.119	0.830	0.027	0.507	0.083	0.807	0.033

Sum of all VOCs	0.093	0.213	0.848	0.033	0.148	0.467	**0.045**	**0.246**	**0.013**	**0.303**	**0.047**	**0.267**

## Abbreviations

CI: Confidence interval
CFU: Colony forming unit
Eo/B: Eosinophil/basophil
ETS: Environmental tobacco smoke
GM-CSF: Granulocyte macrophage colony-stimulating factor
IL: Interleukin
IQR: Interquartile range
LINA: Lifestyle and Environmental Factors and Their Influence on Newborns Allergy Risk
NAMNC: Nonadherent mononuclear cell
OR: Odds ratio
PBMC: Peripheral blood mononuclear cell
*R*: Spearman's rank correlation coefficient
VOC: Volatile organic compound.
