# Supplementary material for: The LINA Study: Higher Sensitivity of Infant Compared to Maternal Eosinophil/Basophil Progenitors to Indoor Chemical Exposures
Source: J Environ Public Health. 2016 May 26;2016:5293932. doi: 10.1155/2016/5293932 (PMC4899584; doi:10.1155/2016/5293932)
Supplement: Supplementary file 1 — Supplementary data provide detailed information about the data collection by questionnaires according to respiratory outcomes as well as lifestyle and environmental conditions. Further, the measurement of urinary cotinine concentration is described. Table A.1 provides relevant case numbers for analyses of indoor chemical exposures and Eo/B CFUs of mothers and infants. Figure A.1 provides an overview about resulting N-numbers for Eo/B CFU analyses within the LINA study. [file 5293932.f1.pdf]

Supplementary data

The LINA study: Higher sensitivity of infant compared to maternal eosinophil/basophil progenitors to indoor chemical exposures

Friederike Hoernig[*,†], Tibor Kohajda PhD[‡], Stefan Roeder PhD[§], Gunda Herberth PhD[*], Martin von Bergen PhD[‡,ǁ,¶], Michael Borte MD[**], Ulrike Diez MD[**], Ulrike Rolle-Kampczyk PhD[‡], Jan-C. Simon MD[††], Judah A. Denburg MD[‡‡], Irina Lehmann PhD[* §§] & Kristin M. Junge PhD[* §§]

[§§] Both authors contributed equally to this work

[*] Helmholtz Centre for Environmental Research – UFZ, Leipzig, Department of Environmental Immunology, Permoserstraße 15, 04318 Leipzig, Germany

[†] LIFE – Leipzig Research Center for Civilization Diseases, University of Leipzig, Philipp-Rosenthal-Straße 27, 04103 Leipzig, Germany

[‡] Helmholtz Centre for Environmental Research – UFZ, Leipzig, Department of Metabolomics, Permoserstraße 15, 04318 Leipzig, Germany

[§] Helmholtz Centre for Environmental Research – UFZ, Leipzig, Core Facility Studies, Permoserstraße 15, 04318 Leipzig, Germany

[ǁ] Helmholtz Centre for Environmental Research – UFZ, Leipzig, Department of Proteomics, Permoserstraße 15, 04318 Leipzig, Germany

[¶] Aalborg University, Department of Biotechnology, Chemistry and Environmental Engineering, Niels Jernes Vej 10, 9220 Aalborg East, Denmark

[**] Children's Hospital, Municipal Hospital "St. Georg", Academic Teaching Hospital of the University of Leipzig, Delitzscher Straße 141, 04129 Leipzig, Germany

[††] University Hospital of Leipzig, Department of Dermatology, Venereology and Allergology, Philipp-Rosenthal-Straße 23, Haus 10, 04103 Leipzig, Germany

[‡‡] McMaster University, Department of Medicine, Division of Clinical Immunology and Allergy, 1200 Main Street West, L8N 3Z5, Hamilton, ON, Canada

Corresponding author:

Dr. Irina Lehmann, Helmholtz-Centre for Environmental Research – UFZ, Department of Environmental Immunology, Permoserstrasse 15, 04318 Leipzig, mail: irina.lehmann@ufz.de

Supplementary Materials and Methods

Questionnaire data

Ever wheezing was recorded as a parental report of symptoms (‘Did your child suffer from whistling / wheezy respiration during the last twelve months?’) with/without the necessity of medical treatment (‘If your child suffered from a whistling/wheezy respiration or cough, was it treated with drugs or by inhalation?’). Recurrent wheezing was recorded as parental report of whistling/wheezy respiration in more than two periods within the second year of life. Bronchitis as well as obstructive bronchitis were recorded as physician diagnoses (‘Has a doctor diagnosed your child with bronchitis/obstructive bronchitis in the last 12 months?’).
Exposure to ETS was assessed as smoking frequency at home (‘Did you or anybody else smoke inside your dwelling?’). Answering this question as ‘(almost) daily’, ‘once a week or more’ or ‘occasionally’ was defined as exposure to ETS in the subsequent analyses and ‘never’ as no exposure to ETS in the dwelling, respectively. Furthermore, the numbers of smoked cigarettes per day in the dwelling (‘How many cigarettes per day were smoked by the mother/father/anybody else in your dwelling?’) was considered. In the subsequent analyses the sum of smoked cigarettes per day of all persons living in the dwelling was used for correlations.
Exposure to disinfectants was assessed by the frequency of their usage in the dwelling (‘How often did you use disinfectants in the household?’). Answering this question as ‘once a week or more’, ‘once a month or more’ or ‘occasionally’ was defined as exposure to disinfectants in the subsequent analyses and ‘never’ as no exposure to disinfectants in the dwelling, respectively.

Analyses of urinary cotinine concentration

Cotinine was analyzed in the urine of the mothers at the child’s first birthday. After dichloromethane extraction and chromatographic separation with a Chromolith Speed ROD

column (Merck, Darmstadt, Germany) cotinine was determined using turbo ionspray ionization on the LC/MS/MS device API 4000 (Applied Biosystems, Darmstadt, Germany). Analysis of creatinine as a measure for individual urinary dilution was used for standardization of the metabolite.

Supplementary Tables

Table A.1 Number of relevant cases for analyses of indoor chemical exposures and IL-3, IL-5 or GM-CSF stimulated Eo/B CFUs of mothers and infants. Data resulting from sub-groups N<5 (cases in brackets) are not shown in our main document.

	**Eo/B CFUs mother N=66**			**Eo/B CFUs child N=68**		
	**IL-3**	**IL-5**	**GM-CSF**	**IL-3**	**IL-5**	**GM-CSF**
	N=63	N=36	N=11	N=67	N=66	N=56
**Exposure to ETS in dwelling**[*]						
yes	6	(2)	(2)	7	7	7
no	56	(34)	(9)	59	58	48
**Number of smoked cigarettes/day in dwelling**						
>0	6	(2)	(2)	7	7	7
0	56	(34)	(9)	59	58	48
**Usage of disinfectant**						
yes	37	20	5	40	40	36
no	24	15	6	25	23	18
**VOCs**[†]	63	36	11	67	66	56

[*] yes = (almost) daily, once a week or more or occasionally; no= never
[†] N may differ due to missing data

Supplementary Figures

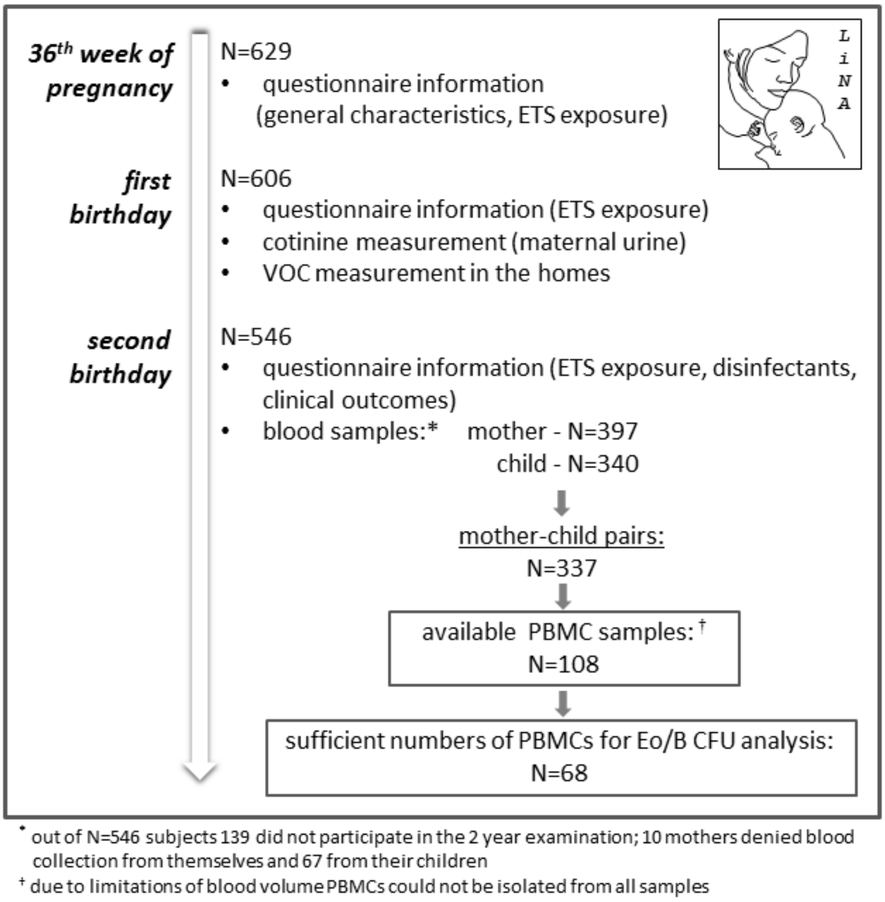

Figure A.1 Relevant N-numbers for Eo/B CFU analysis within the LINA study. Overview about relevant N-numbers and selected information/data from the LINA study which are used in the current paper. Questionnaire information always refers to the previous 12 months.
